# Diffusion Abnormality in Temporal Lobe Epilepsy Patients With Sleep Disorders: A Diffusion Kurtosis Imaging Study

**DOI:** 10.3389/fpsyt.2022.885477

**Published:** 2022-05-26

**Authors:** Min Guo, Boxing Shen, Jinhong Li, Xiaoqi Huang, Jie Hu, Xiaocheng Wei, Shaoyu Wang, Ruohan Yuan, Chengcheng He, Yanjing Li

**Affiliations:** ^1^Department of Radiology, Yanan University Affiliated Hospital, Yanan, China; ^2^MR Research China, GE Healthcare, Beijing, China; ^3^MR Scientific Marketing, Siemens Healthineers, Shanghai, China

**Keywords:** diffusional kurtosis imaging (DKI), temporal lobe epilepsy (TLE), sleep disorders, sleep questionnaires, abnormal white matter structure

## Abstract

**Background:**

Patients with temporal lobe epilepsy (TLE) frequently complain of poor sleep quality, which is a condition that clinicians are typically neglecting. In this study, Epworth Sleepiness Scale (ESS), Pittsburgh Sleep Quality Index (PSQI), and Athens Insomnia Scale (AIS) were used to assess the sleep status of patients with temporal lobe epilepsy (TLE). Simultaneously diffusion kurtosis imaging (DKI) was applied to examine the white matter microstructure abnormalities in patients with TLE and sleep disorders.

**Methods:**

TLE patients who have been diagnosed in the cardio-cerebrovascular ward of the Yanan University Affiliated Hospital from October 2020 to August 2021 were recruited. Finally, 51 patients and 30 healthy controls were enrolled in our study, with all subjects completing the sleep evaluation questionnaire and undergoing a DKI examination. Using independent sample *t*-test, analysis of variance (ANOVA), and Mann-Whitney U test to compare groups.

**Results:**

Thirty patients (58.82%) complained of long-term sleep difficulties. The overall differences among the evaluation of AIS, ESS, and PSQI are significant (*P* = 0.00, *P* = 0.00, *P* = 0.03). The scores of AIS, ESS in Left and Right-TLE (L/R-TLE) with sleep disorders, as well as PSQI in L-TLE, are statistically higher than the control group (*P* = 0.00, *P* = 0.00, *P* = 0.00, *P* = 0.00, *P* = 0.02). L-TLE with sleep disorders showed decreased MK on affected sides (*P* = 0.01). However, statistical differences in MD and FA have not been observed (*P* = 0.34, *P* = 0.06); R-TLE with sleep disorders showed significantly decreased MK and increased MD on affected sides (*P* = 0.00, *P* = 0.00), but FA's statistical difference has not been observed (*P* = 0.20).

**Conclusions:**

TLE patients with sleep disorders have different DKI parameters than individuals who do not have sleep issues. During this process, the kurtosis parameter (MK) was more sensitive than the tensor parameters (MD, FA) in detecting the patient's aberrant white matter diffusion. DKI may be a better choice for *in vivo* investigation of anomalous craniocerebral water diffusion.

## Introduction

The most prevalent intractable focal epilepsy is temporal lobe epilepsy (TLE), which is frequently linked with hippocampal sclerosis (HS) (1). Moreover, the location of TLE tissue damage followed a specific anatomical and functional pattern, with the sections directly or indirectly related to the medial temporal lobe being the most affected (2). Seizures affect patients' cognitive abilities and quality of life (3) and negatively affect sleep structure. Previous studies discovered that poor sleep quality would affect peoples' memory formation and life wellbeing (4). Although the interaction between seizures and sleep issues is still under research, sleep disorders have become a common but often overlooked problem in patients with epilepsy by neurologists. Sleep disorders are common comorbidities in epilepsy, such as obstructive sleep apnea syndrome (OSA), restless legs syndrome, and chronic insomnia. Sleep deficit, poor sleep quality, and daytime sleepiness are common complaints among clinical TLE patients (5). According to statistics, about 50% of patients have chronic insomnia (6).

Early identification of the comorbidity can help ameliorate patients' epilepsy burden and improve their quality of life. Yaranagula (7) found that although surgery can control the frequency of seizures, the sleep quality of the patients did not improve. The sleep quality scores before and after surgery were lower, so the purpose of epilepsy treatment should not be limited to controlling the frequency, but also to treat the disease on the adverse effects on the patient, such as sleep, are minimized. Rapid eye movement (REM) has a protective effect on seizures (8). Previous studies have used polysomnography (PSG) to investigate the sleep structure of TLE patients and compared the sleep status of patients without seizures during the day with the patients after seizures, finding that nighttime REM sleep was significantly reduced after seizures and that reductions were more pronounced after nighttime seizures than daytime seizures, patients also experienced significantly lower sleep efficiency, and the effect of L-TLE on REM was more significant after nighttime seizures than daytime seizures (9).

The anomalous diffusion of cerebral water molecules was discovered in researches that used diffusion tensor imaging (DTI) to examine patients with primary insomnia (PI) (10). Jensen first introduced diffusional kurtosis imaging (DKI) in 2005, which expands traditional DTI by estimating the kurtosis of the water diffusion probability distribution function. DKI can provide both kurtosis parameters and tensor parameters. Many researchers have used DKI to assess microscopic white matter abnormalities in patients with various types of epilepsy using the region of interest (ROI), voxel analysis, and automated fiber quantification (AFQ) and have discovered abnormal brain regions and extensive anomalies in brain networks (1, 11, 12). Nonetheless, this approach has hardly been used in studies to identify subtle structural abnormalities in TLE patients with sleep disturbances. Furthermore, in clinical work, sleep questionnaires are frequently used to examine the subjective sleep experience of TLE patients.

Our purpose is to use the DKI to analyze microstructural abnormalities in white matter in TLE patients with subjective sleep issues and to measure patients' subjective sleep quality by delivering sleep questionnaires. We also looked into the differences in sleep scores between sick and healthy controls.

## Subjects and Methods

### Participants

The local institutional review board of the Yanan University Affiliated Hospital approved this prospective study (number: YAS-S01-202106001), and informed consent was obtained from all participants. From October 2020 to August 2021, patients suspected of having unilateral TLE were recruited for this study. Inclusion criteria were as follows: (1) in line with the International League Against Epilepsy (ILAE) and the International Bureau for Epilepsy (IBE) (13) diagnostic criteria for TLE; (2) younger than 50 years old, since previous research have suggested that adults over 50 had different sleep habits than younger people (14); (3) had no seizures within 24 h before MR examination and (4) had complete DKI-MRI data and sleep status data available. Of the 57 patients initially enrolled, 6 were excluded (2 with obvious image artifacts, 1 with concomitant white matter lesions grade III, 1 is encephalitis secondary epilepsy, and 2 with concomitant traumatic brain injury). Finally, 51 patients with unilateral TLE were enrolled. Another 30 healthy volunteers with no neurological abnormalities were also recruited to form a control group.

**Figure 1:**
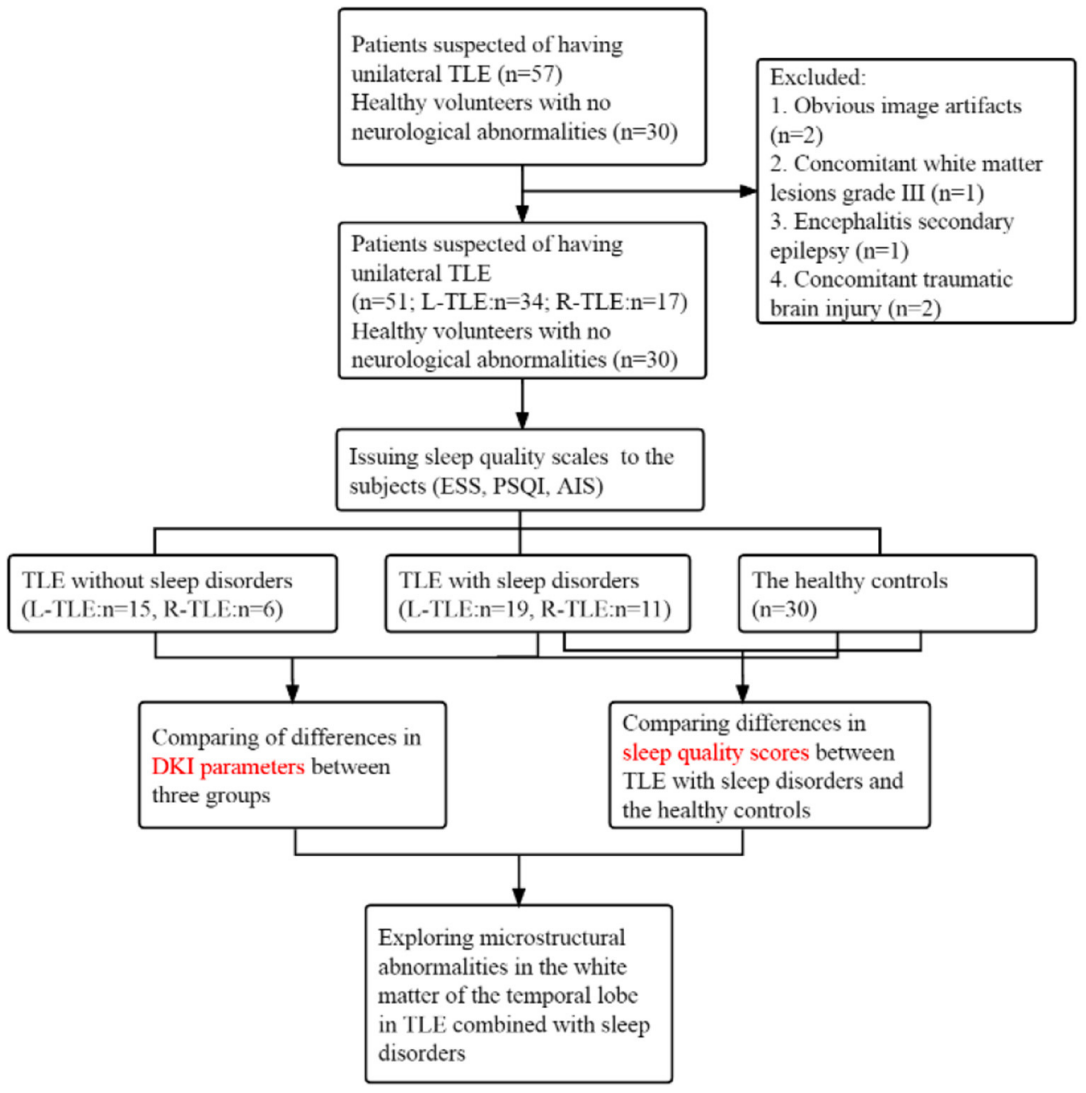
A flow chart is used to show the implementation method of the study.

Neurologists referred to the International Classification of Sleep Disorders-third edition (ICSD-3) and screened patients for underlying sleep disorders, such as insomnia, somnolence, sleep-related breathing disorders or movement disorders (restless legs syndrome), etc. (15), based on their main complaints. Then allocated subjects to 3 subgroups, consisting of TLE patients with sleep disorders, without sleep disorders and the control group. A flow chart is shown in Figure 1.

### Sleep Questionnaires Evaluation

#### Epworth Sleepiness Scale

Excessive daytime sleepiness (EDS) is described as a recent three-month inability to stay awake during the primary awake part of the day. The Epworth Sleepiness Scale (ESS) (16) is a questionnaire that assesses a person's daytime sleepiness. Participants were asked to rate their chances of nodding off in various situations. It can determine the likelihood of dozing off in eight situations that we encounter daily, which spans from 0 (never dozing off) points to 3 (frequently dozing off). Previous research (17) validated the construct validity and internal consistency of the score. An ESS score of more than ten is seen as abnormal (7, 18).

#### Pittsburgh Sleep Quality Index

The Pittsburgh Sleep Quality Index (PSQI) (19) was compiled in 1989 to assess sleep quality during the previous month. It consists of 19 self-evaluated questions and five roommate-evaluated questions, with the last five items being used solely for clinical data. These 19 self-assessment questions look at a variety of sleep-related characteristics, such as sleep length and latency estimates, as well as the frequency and severity of specific sleep-related issues. These 19 items are separated into seven components, and the scores from the seven components are summed together to provide a PSQI score (0–21). The higher the score, the poorer the sleep quality. The sleep quality scale measures subjective sleep quality, sleep latency, and sleep length, among other things. A PSQI score of more than five is considered abnormal (7).

#### Athens Insomnia Scale

The Athens Insomnia Scale (AIS) (20) is an eight-item self-assessment of sleep status with a total score of 0–24, with 1–5 questions evaluating sleep status and 6–8 items evaluating daytime mental state, an AIS score of more than six are considered abnormal (21). The Chinese version (22) of AIS has been proven to be reliable and effective in adolescents and adults.

### Magnetic Resonance Imaging Acquisition and Data Analysis

Images acquisition were performed on a 3T MRI scanner (Magnetom Verio, Siemens Healthineers, Erlangen, Germany) equipped with an 8-channel head coil. The conventional MRI protocols included the following sequences: axial T1-weighted, axial T2-weighted, and axial FLAIR images. The detail parameters about DKI imaging were: TR = 11000 ms, TE = 96 ms, field- of- view = 230 × 230 mm^2^, matrix size = 100 × 100, scanning slice thickness = 2 mm, b = 0, 1,000 and 2,000 s/mm^2^, using 30 different diffusion coding directions totally.

**Figure 2:**
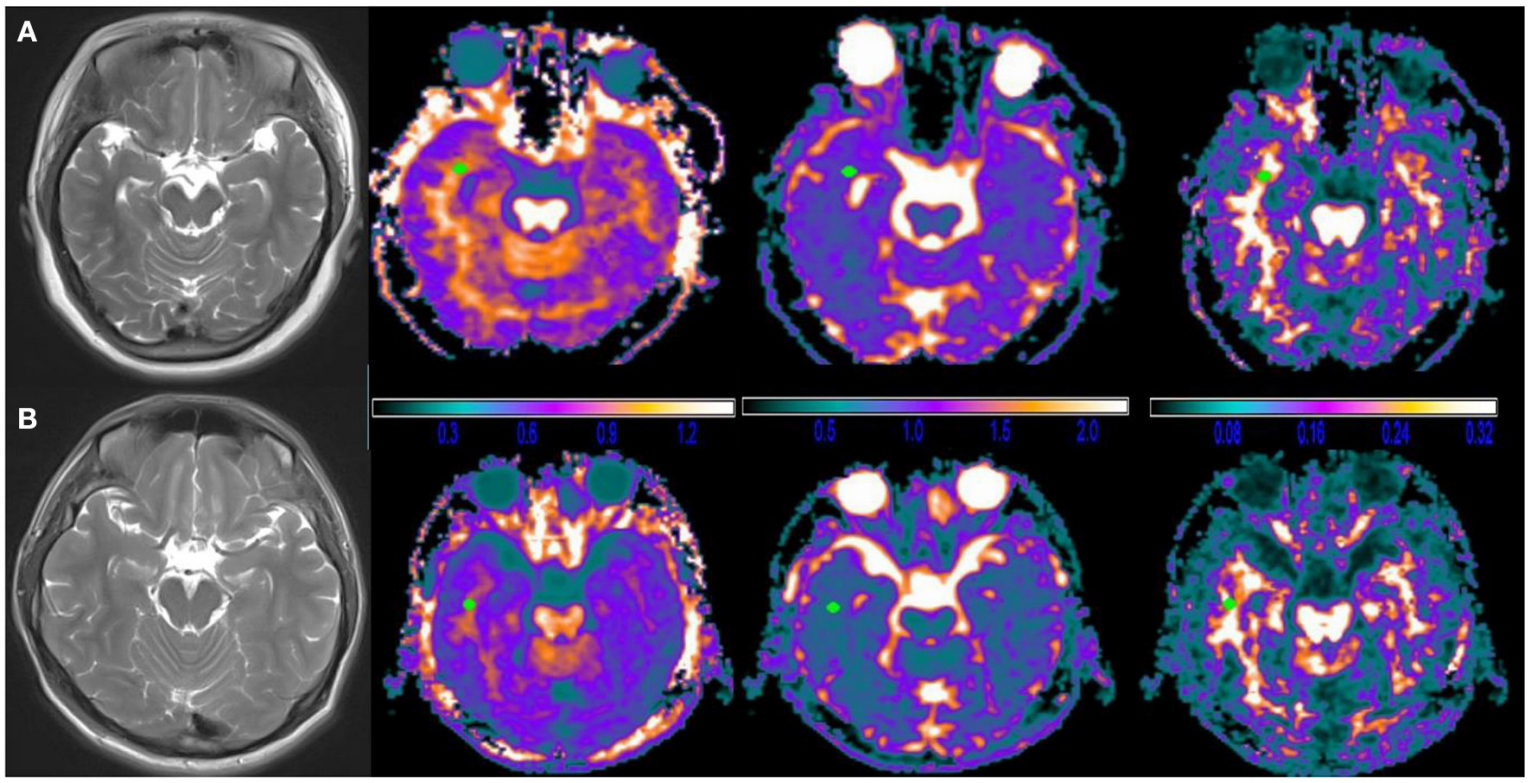
Typical diffusional kurtosis imaging (DKI)-derived parametric maps in a case brain. **(A)** Female, 27 years old, R-TLE with sleep disorder, **(B)** Male, 35 years old, R-TLE without sleep disorder; from left to right are T2WI images, mean kurtosis (MK) maps, mean diffusivity (MD) maps and fractional anisotropy (FA) maps. No clear abnormalities were seen on T2WI images and DKI images in both patients, which could be detected by measurement of DKI parameters. Note: the unit of MD is μm^2^/ms, and FA, MK are dimensionless.

The images were double-blind analyzed by two neuroimaging physicians who have worked for over 10 years and the average of the two was taken as the final result. The diffusional kurtosis estimator software (https://www.nitrc.org/projects/dke/Version2.6) was used to analyze raw diffusion images and calculate DKI's parametric maps, specifically maps of mean diffusivity (MD), fractional anisotropy (FA), and mean kurtosis (MK). Figure 2 shows a representation of DKI's major parametric maps. Using the T1WI image as a reference, one circular ROI was drawn in the white matter of the temporal lobe on the affected side of patients and matched side of control group through MRIcron software (https://www.nitrc.org/projects/mricron/Version1.0.20190902), avoiding the sulci, split-brain, and ventricle. The ROI would be no <25 mm^2^, and the average would be taken by measuring at the same point on three consecutive levels of MK, MD, and FA graphs (23).

### Statistical Analysis

SPSS software was used to analyze the data (version 20.0). Continuous variables utilize mean±standard deviation (m±SD), while categorical variables use frequency. An independent sample *t*-test, analysis of variance (ANOVA), and Mann-Whitney U test were used to compare between groups, depending on the normality of the data. *Post-hoc* pairwise comparisons were performed using *LSD*-*t* test. The Chi-square test and Fisher-Freeman-Halton test were used to study the association between categorical variables. A *P* < 0.05 is considered significant.

## Results

### Demographic and Clinical Characteristics of Subjects

After screening, 51 TLE were finally enrolled (23 males and 28 females). Video EEG revealed unusual temporal lobe discharge on one side, including 34 cases of Left-TLE (L-TLE) and 17 cases of Right-TLE (R-TLE). Table 1 shows demographic information and clinical traits. Thirty patients (58.82%) complained of long-term sleep difficulties, while 21 thought sleep quality was satisfactory. Five patients (9.80%) did not take any medications; the others took the single antiepileptic therapy (AED).

**Table 1:** The demographic and clinical characteristics in subjects of the study.

**Characteristics**	**L-TLE (*n* = 34)**	**R-TLE (*n* = 17)**	**HC (*n* = 30)**	***F*/*χ^2^*/*z***	***P* value**
Age (years)	23.52 ± 11.10	27.45 ± 8.51	28.52 ± 11.53	1.29	0.28^a^
Duration (years)	3 (0.42,8)	1.2 (1.0,6.7)	/	−1.30	0.19^b^
Gender (male/female)	16/18	7/10	11/19	0.71	0.70^c^
Sleep disorder (with/not)	19/15	11/6	0/30	28.41	0.00^c^
**Type of AED:**
Levetiracetam	17	6	/	1.37	0.71^c^
Lamotrigine	6	5	/		
Valproate sodium	8	4	/		
No medication	3	2	/		

*L-TLE, Left-TLE; R-TLE, Right-TLE; HC, The healthy control group*.

*^a^Analysis of variance (ANOVA) ^b^Mann-Whitney U-test ^c^Chi-square test*.

### Results of Self-Evaluation Sleep Questionnaire

**Figure 3:**
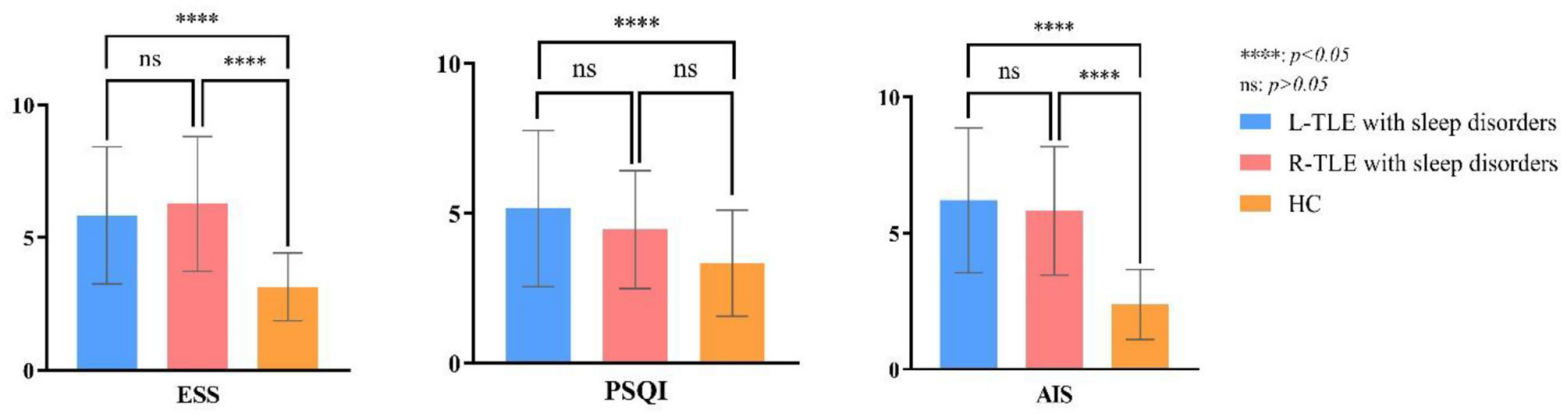
*Post-hoc* multiple comparison in sleep scores of TLE with sleep disorders and the healthy controls.

Table 2 compares the overall differences in AIS, ESS, and PSQI scores between TLE with sleep disorders and the control group. The AIS, ESS, and PSQI total differences are statistically significant (*P* = 0.00, *P* = 0.00, *P* = 0.03). Figure 3 shows the pairwise comparison: the scores of L/R-TLE in AIS, ESS, and PSQI in L-TLE are statistically higher than the control group (*P* = 0.00, *P* = 0.00, *P* = 0.00, *P* = 0.00, *P* = 0.02). Although the difference in PSQI between R-TLE and the control group was not statistically significant, the mean value was higher than the control group. The number of patients with abnormal scores (AIS≥6, ESS≥10, and PSQI>5) was few, and no statistical difference was detected.

**Table 2:** The score of self-evaluation sleep questionnaire in subjects of the study.

**Items**	**AIS(score)**	**ESS(score)**	**PSQI(score)**	**Number**		
				**score of AIS≥6**	**score of ESS≥10**	**score of PSQI>5**
L-TLE (*n* = 19)	6.21 ± 2.66	5.84 ± 2.58	5.16 ± 2.60	10	2	5
R-TLE (*n* = 11)	5.81 ± 2.35	6.27 ± 2.53	4.46 ± 1.97	6	1	3
HC (*n* = 30)	2.38 ± 1.28	3.15 ± 1.31	3.34 ± 1.77	0	0	0
*F*/*χ^2^*	18.79	3.62	3.96	0.25
*P* value	0.00^a^	0.00^a^	0.03^a^	1.00^b^

^a^*Analysis of variance (ANOVA) ^b^Fisher-Freeman-Halton test (n <40)*.

### MK, MD and FA Values in White Matter of the Temporal Lobe on Subjects

**Figure 4:**
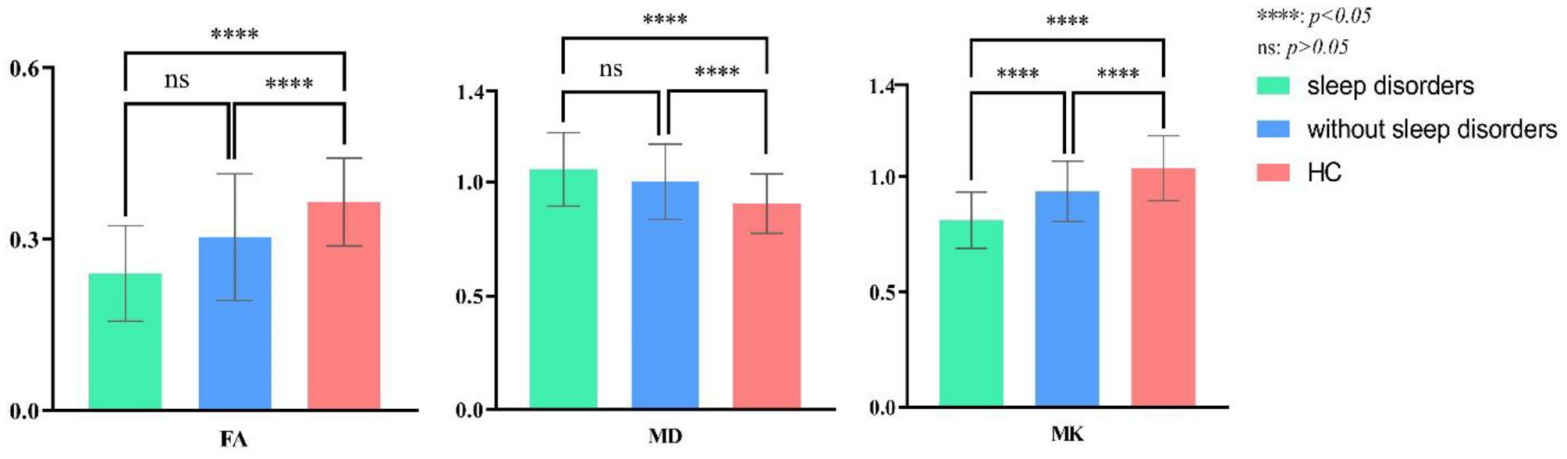
*Post-hoc* multiple comparison in DKI parameters of L-TLE with or without sleep disorders and the healthy controls.

Nineteen combined with sleep disorders and 15 without in L-TLE, as illustrated in Table 3. We compared the DKI parameters in three groups: L-TLE with sleep disorders, L-TLE without sleep disorders, and the control group. We manually delineated the ROI and quantified the MK, MD, and FA values on the afflicted side (left) of a patient and matched the side of the control group (left). Table 3 and Figure 4 demonstrate the results.

**Table 3:** ANOVA of DKI parameters between L-TLE with or without sleep disorders and the healthy controls.

**DKI parameters**	**L-TLE (*****n*** **=** **34)**		
	**MK**	**MD**	**FA**
With sleep disorder (*n* = 19)	0.8112 ± 0.12	1.0550 ± 0.16	0.2401 ± 0.08
Without sleep disorder (*n* = 15)	0.9374 ± 0.13	1.0011 ± 0.16	0.3041 ± 0.11
HC-L (*n* = 30)	1.0299 ± 0.15	0.9049 ± 0.13	0.3655 ± 0.08
*F*	15.61	6.33	13.32
*P* value	0.00	0.00	0.00

The overall difference in MK, MD, and FA between these three groups was statistically significant (*P* = 0.00). The FA, MK of TLE decreased, and the MD increased compared to the control group, regardless of whether sleep disturbance was combined (FA: *P* = 0.00, *P* = 0.01; MD: *P* = 0.00, *P* = 0.04; MK: *P* = 0.00, *P* = 0.03). Especially MK was significantly lower in the sleep disorder group than without sleep disorder (*P* = 0.01). FA was also lower, though not statistically significant (*P* = 0.06). We did not observe a significant difference in MD between the sleep disorder group and those without (*P* = 0.30).

**Figure 5:**
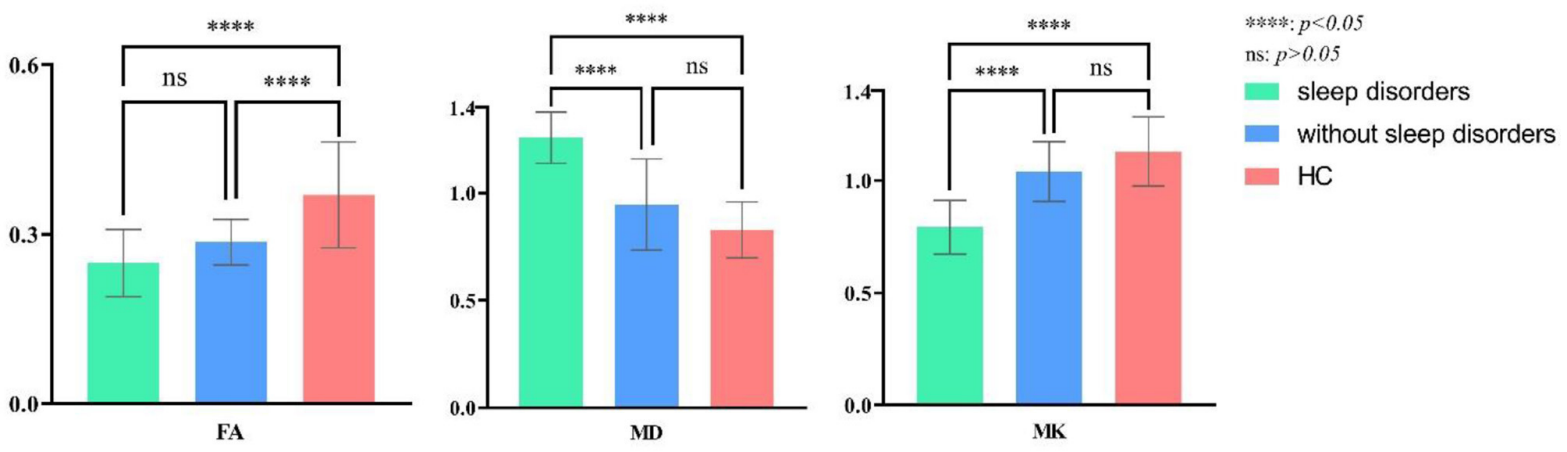
*Post-hoc* multiple comparison in DKI parameters of R-TLE with or without sleep disorders and the healthy controls.

In R-TLE, eleven combined with sleep disorders and six without, as illustrated in Table 4. As with L-TLE, we compared DKI parameters between these three groups in the patient's afflicted side (right) and matched side of the control group (right): R-TLE with sleep disorders, R-TLE without sleep disorders, and the control group. Table 4 and Figure 5 demonstrate the results.

**Table 4:** ANOVA of DKI parameters between R-TLE with or without sleep disorders and the healthy controls.

**DKI parameters**	**R-TLE (*****n*** **=** **17)**		
	**MK**	**MD**	**FA**
With sleep disorder (*n* = 11)	0.7924 ± 0.12	1.2568 ± 0.11	0.2494 ± 0.06
Without sleep disorder (*n* = 6)	1.0401 ± 1.13	0.9459 ± 0.21	0.2863 ± 0.04
HC-R (*n* = 30)	1.1312 ± 0.15	0.8280 ± 0.13	0.3701 ± 0.09
*F*	22.35	39.24	9.81
*P* value	0.00	0.00	0.00

The overall difference in MK, MD, and FA between these three groups was statistically significant (*P* = 0.00). The FA, MK of TLE with sleep disorders reduced, and the MD increased than that of the control group (FA: *P* = 0.00; MD: *P* = 0.00; MK: *P* = 0.00). There were only six individuals in R-TLE without sleep disorders, so only FA was found to be reduced in without sleep disorder group than the control group (*P* = 0.00), and we did not found difference in MK and MD between them (MD: *P* = 0.06; MK: *P* = 0.17).

The sleep disorder group found significantly higher MD and lower MK patients (MD: *P* = 0.00; MK: *P* = 0.00). The difference in FA between the sleep disorder group and those without has not been observed (*P* = 0.39).

## Discussion

Our study's combination of sleep disorders with DKI is a significant advantage, which previous investigations hardly used. Another advantage is the homogeneity of subjects; we controlled for factors that might affect sleep quality, such as age and the amount of medication.

Mechanisms of the interaction between seizures and sleep have been under investigation. Abnormal electrical discharges between seizures can lead to sleep disruption (6), and the disruption of a patient's normal sleep would exacerbate the seizures. Sleep quality also affects the type and likelihood of a patient's seizures, and the association between sudden unexpected death in epilepsy (SUDEP) and sleep is also gradually being recognized.

Our research grouped the sleep condition of patients with TLE and investigated the microstructure through DKI. Meanwhile, we evaluated the sleep quality scores between patients with sleep disorders and the control group by sleep questionnaires. We discovered that TLE with sleep difficulties are pretty common (58.82% in this study), and the values of DKI's parameters also have anomalies.

The diffusion environment of water molecules is affected in TLE due to injured nerve fiber bundles, disrupted white matter integrity, and proliferation of irregular glial cells. Therefore, differences exist in DKI parameters between TLE and the controls. The preliminary results of the present study are also close to our previous works on the microstructure of TLE. However, we aimed to apply the DKI technique to subgroup further comparisons between comorbid sleep disorders and non-comorbid sleep disorders. We want to obtain more information about comorbidities and support individualized treatment of patients with sleep problems.

TLE patients frequently experience insomnia, poor sleep quality, and excessive daytime drowsiness. The prescription of AEDs mainly focused on reducing seizures, and it is easy to overlook other mental and sleep disorders that patients may be experiencing. Our study only included individuals who used one AED to minimize the effects of AEDs on sleep. Drugs have a significant impact on the sleep quality of PWE, prior investigations have reported that combining numerous AEDs can result in a decrease in sleep quality in patients (24).

Twenty-three patients (50%) utilized Levetiracetam in our investigation. Studies (25) have shown that large-dose levetiracetam use caused excessive drowsiness during the day. Patients with sleep disorders had a higher ESS score could be related to their medication. Furthermore, daytime sleepiness in patients may be related to other multiple factors, such as nocturnal seizures, sedative effects of AEDs, and sleep deprivation at night. However, the number of abnormal ESS scale scores was lower, probably because excessive daytime sleepiness was more common in frontal lobe epilepsy than TLE (26). Lamotrigine and valproate sodium were the other two medicines the patients took in this study. Foldvary (27) compared lamotrigine to several older antiepileptic medicines and concluded that lamotrigine users rarely have significant daytime sleepiness or night sleeplessness. As Foldvary's article indicates, valproate sodium also has no substantial effect on sleep structure (28).

White matter plays a vital role in regulating brain activity and the coupling between brain regions and behavioral regulation (29). Previous studies have found that the proportion of fiber bundles in the left striatum and hippocampus of insomnia patients reduced through DTI technology (30), which may mean the abnormal reduction of white matter fiber tracts in insomnia patients. To further explore the microstructure abnormalities of TLE with sleep disorders, our study used DKI for patients' brain imaging and analysis. Compared with DTI, DKI can describe higher-order diffusion dynamics and more complex diffusion distribution. DKI could detect complex and crossed white matter fiber bundles and provide more comprehensive quantitative parameters, which will help improve the disease detection rate of white matter abnormalities (1). Although the number of studies related to abnormal white matter structure in sleep disorders is lacking, it has confirmed that abnormal changes in microstructure and brain networks exist in the brain of this group, and this abnormality spreads from the limbic system to the entire brain (31).

Numerous previous studies have demonstrated that sleep plays an essential role in the brain. Neuroimaging found sleep disturbances associated with functional deficits as measured by fMRI or atrophy of the cerebral cortex, and the integrity of the white matter microstructure may underlie these associations. Poor sleep may disrupt axonal integrity and degenerate white matter, but white matter pathology may also precede sleep disturbance (32).

Voldsbekk et al. (33) combined spherical mean technique (SMT) and diffusion imaging to inquire into differences between sleep deprivation and normal sleep-wake-cycle (SWC) group. He discovered that sleep deprivation was associated with extensive white matter changes and had an abnormal intracranial diffusion coefficient. Vyas (34) utilized DKI to brain microstructural changes in OSA and detected abnormalities in kurtosis parameters in several brain regions of the patients. They indicated that kurtosis parameters are more sensitive to abnormalities shown at the microcosmic structure level before detectable abnormalities appear in conventional MRI or other imaging modalities.

As a classic parameter of DKI, MK is related to the microstructure changes of many diseases and is sensitive to more subtle brain changes. The increase in the myelin sheath of white matter fibers, dense accumulation of axons and fiber bundles, and decreases in the permeability of the axon membrane caused increases in kurtosis parameters. MK decreased in the sleep disorder comorbidity group in our study. It could be due to a net loss of microscopic tissue complexity caused by damage to the myelin barrier and other microscopic cell structures in these areas, such as the loss of nerve synapses. Which results in decreased cell connections, decreased tissue integrity, and increased extracellular gap (35), and is also consistent with pathological denervation (1). Epilepsy activity and long-term parasomnia may make water molecules in the brain more susceptible to spreading toward synchrony. In an immunohistochemistry investigation of Huntington's disease (36), the number of fibers that can be stained and arranged orderly reduced in regions with decreased kurtosis values.

The present study differs from Tummala's study on OSA (37), which found increased kurtosis parameters in several intracranial regions. They suggested that the hypoxic and ischemic damage caused by OSA may cause swelling of neurons and axons, leading to acute axonal and myelin tissue damage. This study also proposed that the mechanism for the increased kurtosis parameter in acute disease may be the incremental extracellular fluid due to degeneration of axons and myelin sheaths, the more pronounced the non-Gaussian nature of water molecule diffusion. In contrast, in chronic disease, for instance, epilepsy, the diffusion of water molecules tends to be more Gaussian in distribution due to the decrease in axons, myelin, neurons, and glial cells and the increase in extracellular gaps.

MD variations linked to the activity dynamics of epileptic seizures according to the previous research (38, 39): in the hyper-acute phase after protracted convulsions or status epilepticus, MD decreases due to cytotoxic edema. MD gradually develops during the sub-acute attack period (~5 days), which is linked to angioedema, while the chronic phase of neuron loss and gliosis leading to an increase in interstitial water content will further lead to an increase in MD.

In our study, the MD of TLE with sleep disorders also tended to higher than patients without sleep disorders. This difference is significant in R-TLE, which may also be consistent with reports of increased MD in the sub-acute state because all patients included in this study had no seizures within 24 h before the scanning. The finding also supports the notion of extensive axonal and neuronal damage. Our results were consistent with research on Rapid eye movement (REM) sleep behavior disorder (RBD), which found the increased MD value in many brain regions (40).

FA is the most often used parameter in daily work and scientific research in neuroimaging and represents water molecules' degree of anisotropic diffusion. The degree of dispersion more noticeable, the bigger the FA. Although the difference in FA in our study was not significant, a downward trend can still be seen, particularly in L-TLE. A decrease in FA reflects the blockage of water molecule transport in the lesion area. The variations in axon density and the integrity of the myelin membrane frequently disrupted water diffusion, the transient increase in diffusion rate generated by the start of angioedema may enhance the probability of water molecules encountering the barrier (41, 42). Our results are similar to Kang's article. He discovered differences in white matter tract spread measures on white matter connections in the left thalamus and inferior frontal gyrus in insomniacs. Insomniacs had compromised white matter integrity, and FA reduced compared with healthy controls. The results may also provide new evidence for decreased connectivity in the thalamic-frontal regions of insomnia patients in functional imaging studies (43). In the future, increasing the sample size may result in more statistically significant findings.

We did not overemphasize the causal relationship between TLE and sleep disorders, which may lead to inappropriate treatment of sleep disorders. Some neurologists may focus on resolving sleep disorders by controlling the primary disease, thus neglecting to correct the patient's sleep problems alone. However, treating the primary disease and sleep disorders should be carried out simultaneously and curing sleep problems also promotes the effectiveness of treatment of the primary disease (15).

However, we still have some limitations: First and foremost, since we have no obtained histology specimens, we must rely on past research to make assumptions. We should collect more longitudinal data from patients to investigate the disease's unique mechanisms. Second, to avoid drug effects on sleep quality, the patients we studied all used only one AED, which may leave out the group of patients treated with a mix of medicines and could have more specific abnormalities in diffusion parameters. Third, our study is a cross-sectional study, and we have not yet obtained a causal relationship from it. So it is unclear whether the abnormality in the observed white matter microstructure is the cause or the result. We hope that future studies with longitudinal designs will identify causal directionality. Fourth, the study's sample size was small (especially in R-TLE), and future continued expansion of the sample size will hopefully lead to more meaningful results.

## Conclusions

To summarize, our research discovered the abnormal DKI parameters in TLE with sleep disorders. The kurtosis parameter (MK) is more sensitive than the tensor parameters (MD, FA) in detecting aberrant white matter diffusion in the patient during this procedure. The DKI properties revealed in our study may represent the underlying pathophysiological mechanism of TLE with sleep disorders, which will need verifying in future investigations.

## Data Availability Statement

The original contributions presented in the study are included in the article/supplementary material, further inquiries can be directed to the corresponding author.

## Ethics Statement

The studies involving human participants were reviewed and approved by Ethics Committee of the Yanan University Affiliated Hospital. Written informed consent to participate in this study was provided by the participants' legal guardian/next of kin.

## Author Contributions

MG and YL proposed the article concept and wrote the manuscript. BS, JH, and JL conducted data collection and analysis. RY and CH conducted the statistical analysis. XW and SW supported the MR technology for this article. XH, MG, and YL supervised the whole research and received funding. All authors have read and approved the final version of the article.

## Funding

The Red Cross Foundation of China funded the study (approval number: XM_HR_ICON_2020_10_07).

## Conflict of Interest

XW was employed by GE Healthcare. SW was employed by Siemens Healthineers. The remaining authors declare that the research was conducted in the absence of any commercial or financial relationships that could be construed as a potential conflict of interest.

## Publisher's Note

All claims expressed in this article are solely those of the authors and do not necessarily represent those of their affiliated organizations, or those of the publisher, the editors and the reviewers. Any product that may be evaluated in this article, or claim that may be made by its manufacturer, is not guaranteed or endorsed by the publisher.
